# Immunohistochemical and morphological characteristics of tissues response to polylactic acid membranes with silver nanoparticles

**DOI:** 10.4317/medoral.23171

**Published:** 2019-12-24

**Authors:** Grigory Demyashkin, Eugenia Kogan, Leonid Borozdkin, Tatyana Demura, Elena Shalamova, Zelimhan Centroev, Irina Ivanova, Margarita Gevandova, Vlada Smirnova-Sotmari, Sergey Kalinin

**Affiliations:** 1 PhD, MD, professor; Department of Pathology. Sechenov University, Moscow, Russia; 2 Member of the European Association of Pathologists; 3 PhD, student at Maxillofacial Surgery Department. Sechenov University, Moscow, Russia; 4 Student at International School "Medicine of the Future”. Sechenov University, Moscow, Russia; 5 Resident at Maxillofacial Surgery Department. Sechenov University, Moscow, Russia; 6 PhD, Head of the Department of Biology, Stavropol State Medial University, Stavropol, Russia; 7 Student at Department of Pathology, Sechenov University, Moscow, Russia

## Abstract

**Background:**

The aim of this research was to study anti-microbial and anti-inflammatory characteristics of silver nanoparticles helping bone structures to recover during late stage of parodontitis, which afterwards will increase the effect of bone regeneration operations.

**Material and Methods:**

We assessed colloid solution-derived silver nanoparticles coating of polylactic acid membrane regarding tissue foreign body response. Thirty eight polylactic acid membranes were implanted intracranially in rabbits – ten unmodified (control group) and twenty eight with silver nanoparticles coating (experimental group). In controls, penicillin was used for infection prophylaxis. Tissue response was assessed by light microscopy and immunohistochemistry (CD3, CD15, CD30) 2 weeks after implantation.

**Results:**

inflammation markers in experimental group were significantly lower than in control group, there were no signs of forming a fibrosis capsule nor infectious signs.

**Conclusions:**

colloid silver solution can be used as a source of nanoparticles for anti-microbial and anti-inflammatory biodegradable membranes’ coating.

** Key words:**Guided bone regeneration, polylactic acid membrane, silver nanoparticles.

## Introduction

Up to date, bone tissue loss is a challenging problem in periodontal disease and maxillofacial surgery (1-3). As a consequence, effective methods to regenerate destroyed bone structure are being actively sought (3-7). In guided bone regeneration, bone-substitute materials can be divided into xenogenic, allogenic, and alloplastic synthetic grafts (8). Barrier membranes of different structure are used to preserve bone grafts from being resorbed, latter being shown to be a significant problem (2,9). Non-degradable barrier membranes has been shown to prevent resorption of bone grafts, however, the ineviTableneed for the re-operation to remove the membrane remains a recognized drawback (2). This problem is solved by using biodegradable materials such as polylactic acid (PLA) membranes. However, such membranes can still lead to a foreign body reaction together with degradation process products causing aseptic inflammation of various intensity. In case of suture line disruption above the reconstruction zone, there is a possibility of infection of the newly regenerated tissue leading to its loss.

In surgical suture materials studies, it has been shown that silver nanoparticles improve biodegradable membranesʼ quality. Silver nanoparticles with their broad range of antimicrobial activity can be considered as an effective alternative to antibiotics (10,11). In recent years, it was also reported that silver nanoparticles in biodegradable membranesʼ coating show anti-inflammatory and anti-fibrotic activity (12). In the present paper, we describe colloid silver nanoparticles as a source of effective anti-microbial and anti-inflammatory biodegradable membranesʼ coating.

The study aimed to assess anti-inflammatory and anti-microbial properties of polylactic acid membranes modified with colloid solution-derived silver nanoparticles. In order to achieve this objective, we assessed: the inflammatory response (infiltrate features, CD3, CD15, CD30); the intensity of fibrosis (fibroblasts, capsule) and the frequency of infectious complications (signs of infection) via the set of histological and immunohistochemically methods.

Materials and Methods 

- Animal model for *in vivo* study:

Experimental research was carried out at Sechenov University, Moscow. 3 – 4 months-old male chinchilla rabbits (n=38) (average body weight 350 – 450 g) were used as models. The animals were kept in a vivarium at 15-hour daylight and at temperature of 22 degrees Celsius. All manipulations conformed to International recommendations upon conduction of biomedical researches with the use of animals and Russian Ministry of Health order № 708н of 23.08.2010 “Concerning Approval of the Rules of Laboratory Practice”.

- Synthesis and characterization of PLA membranes with AgNPs:

Polylactic acid membranes (n=38, 1×1 cm, mass 0,011 mg) were prepared by elecrospinning from polylactic acid fibres (Sigma-Aldrich Co. LLC, USA) solution in hexafluoroisopropanol (Sigma-Aldrich Co. LLC, USA), concentration 100 mg/ml. 28 PLA membranes were subsequently modified by immersion into colloid silver nanoparticles solution NanArgol® (KorolyovFarm LLC, Russia). According to the manufacturer, silver nanoparticles size lies in the range of 0.5 to 3.0 nm whereas their concentration is 0.2 mg/L, measured at 23±2 degrees Celsius via atomic-emissive spectrometer iCAP 6300 Radial View. The major amount of AgNPs are in non-agregated state, which is an important condition for anti-bacterial characteristics of the nanoparticles and for the stability of the colloid solution.

As a result of imbibition of the AgNPs solution into the porous structure of the membranes and AgNPs adsorption on the surface of the polylactic fibres, an antimicrobial zone is formed in the place where the membrane contacts with tissues, which prevents the appearance of undesirable microbiota and at the same time promotes the healing of injuries.

- Treatment of animals with PLA membranes with AgNPs:

The animals were divided into two groups: control group (n=10) treated with PLA membranes without modifications and experimental group (n=28) treated with PLA membranes with colloid silver nanoparticles coating. Penicillin was administered by intramuscular injection in the control group (50 mg/kg b.w., 7 days).

In 38 anaesthetized (Ketamine 100 mg/kg + Xylazine 10mg/kg + Acepromazine 3 mg/kg body weight) rabbits, cranial bone defect was created and a polylactic acid membrane was set in the graft site. Two weeks following the surgery, animals were sacrificed with an overdose of intravenous sodium pentobarbital under deep anesthesia.

- Collection of tissues from rabbits:

The implant with surrounding tissues of the graft site (soft tissues and occipital bone 4×3 cm) was harvested and prepared for the histological analysis.

- Histological examination:

Fixation of the block sections was performed in 10% Neutral Buffered Formalin (NBF) (pH is about 7,2 – 7,4) for 2 weeks with subsequent embedding in paraffin and staining with hematoxylin and eosin and Masson’s trichrome.

Morphometry was performed as follows: neutrophils, red blood cells, lymphocytes, and macrophages were counted in inflammatory infiltrate (in %) in ten high power fields (×400) with mean value and standard deviation calculation.

- Immunohistochemical analysis (IHC):

After deparaffinization and redehydratation of paraffin sections in ethanol series IHC-test was performed automatically in the immunohistostainer Bond-Max (“Leica”, Germany).

To restore the antigenic characteristics of tissue after fixation in formalin heat induction of epitope retrieval (HIER) was conducted. To perform this the glass was heated in citrate buffer (рН=6.0), its being autoclaved (t°+121°С, for 8 min) and symmetrically arranged in the cuvette.

Primary antibodies (bond ready to use reagent) were represented by antibodies to CD3 (clone LN10, NovocastraTM, Great Britain), CD15 (BY87, NovocastraTM, Great Britain), and CD30 (JCM182, NovocastraTM, Great Britain). After that a monitoring study took place for the sake of excluding pseudo-positive and pseudo-negative results. Antibody titer was selected using the antibody diluents solution (1: 100 dilution; cross sections – two).

Secondary antibodies (universal, were purchased from Cell Marque (USA)) containing large quantities of horseradish peroxidase molecules were applied to the sections and incubated in humid chambers for 30 minutes their being washed in Tris-buffer solution between each stage for 10 min.

After application of each reagent slides with cross sections were washed in 0.1 M phosphate buffer solution in a vessel with stirrer. To detect and visualize the reaction 1 – 3 drops of DAB (3,3′-Diaminobenzidine) Substrate Chromogen (Novolink, Leica Biosystems, Great Britain) were added to each reaction section. They were incubated from 30 seconds to 20 min under the microscope control until the dark-brown staining of specific structures became apparent (cytoplasmic reaction). The cell nuclei were then counterstained with Mayers’ hematoxylin, the sections being subject to degradation and then being put into «Aquatex» gel (aqueous mounting agent, «Andwin Science», France).

Samples were analysed under light microscopy («Carl Zeiss Lab.A1», Carl Zeiss, Germany), with digital camera («AxioCam ERc5s», Carl Zeiss Microscopy GmbH, Germany) and appropriate analysis software (ZEN Lite, Germany).

Then, the proportion of immunopositive cells (in %) in 10 fields of view at 400× magnification was calculated: “–” – absence, “+” – weak (1–10% of cells), “++” – moderate (11–50% of cells), “+++” is an intensive (≥51% cell) reaction.

- Statistical analysis

The results were statistically processed with the SPSS 7.5 for Windows statistical software package (IBM Analytics, USA), including the calculation of arithmetic averages with its maximum deviations and standard error. The correspondence of the data to the normal distribution was checked by the Kolmogorov-Smirnov test. The nonparametric Mann-Whitney U-test was used to assess the statistical significance of average differences between the groups. In the absence of a normal distribution, The Wilcoxon signed-rank test was used with *p*<0.05 significance cutoff.

The study was approved by the Ethical Committee of Sechenov First Moscow State Medical University (Protocol № 8; May 12, 2018). All the actions complied with the Declaration of Helsinki (WMA Declaration of Helsinki – Ethical Principles for Medical Research Involving Human Subjects, 64th WMA General Assembly, Fortaleza, Brazil, October 2013).

## Results

- Histological examination

**Figure 1:**
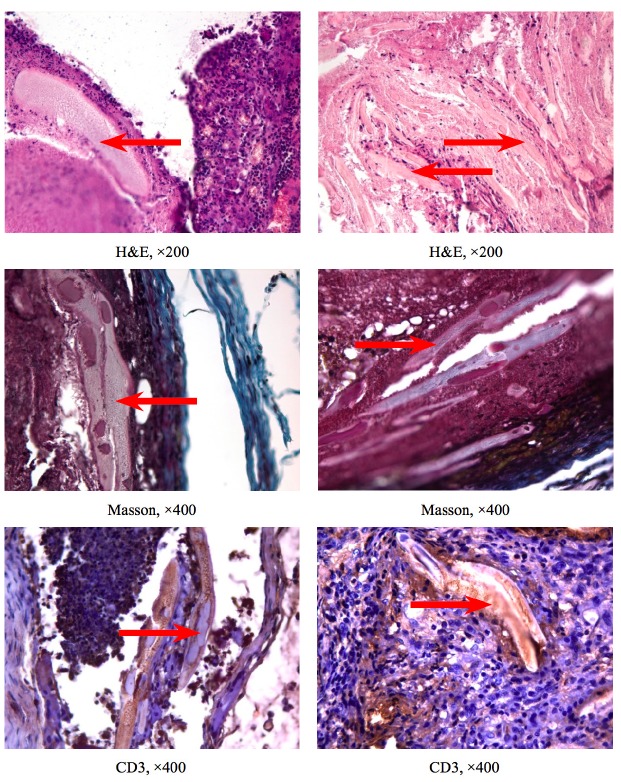
Results of the histological, histochemistry and immunohistochemistry examinations in the control (left) and experimental (right) groups. Staining: H&E, 200− × magnification; staining: Masson, 400− × magnification. Antibodies to: CD3 (lymphocytes); post-staining: hematoxylin, 400− × magnification. Red arrows indicate the membranes.

**Figure 2:**
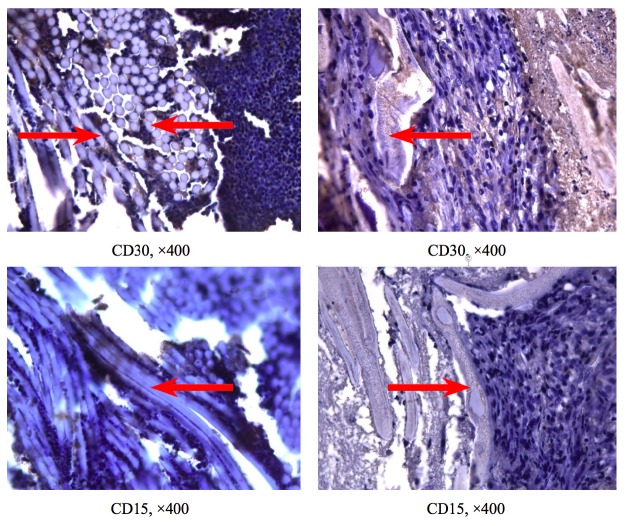
Results of the immunohistochemistry examinations in the control (left) and experimental (right) groups. Antibodies to CD30 (activated lymphocytes), CD15 (neutrophils); post-staining: hematoxylin, 400− × magnification. Red arrows indicate the membranes.

In the control group (n=10), we found the structures of the preserved membrane (20 – 40%), severe chronic inflammation with a predominance of eosinophils, lymphocytes, and macrophages (38.1±5.3%, 18.2±4.8% and 27.3±4.5%, accordingly), single polymorphonuclear leukocytes. On the implant periphery, a connective tissue capsule was formed, consisting of collagen fibers and fibroblasts. One specimen demonstrated fields of cellular detritus. In samples of the experimental group (n=28), we observed varied membrane preservation (5 – 48%), a mild inflammatory reaction with few lymphocytes and macrophages, and membrane replacement by a network of thin collagen fibers and fibroblasts lacking a well-defined capsule formation. Masson’s stain revealed a decrease in the proportion of thick collagen fibers (blue staining) and the presence of elastic fibers (red-yellow staining) compared with the control group (Fig. 1, Fig. 2).

- Immunohistochemistry

The control group (n=10) demonstrated a moderate positive reaction with antibodies to CD3 (17,3±5,2%) and CD30 (14,1±3,6%), including the intercellular matrix (Fig. 1, Fig. 2). CD15 staining showed a weak immunopositive response (3,4±1,2%). In experimental group (n=28), all immunomarkers revealed a weak positive reaction: CD3 (6,5±3,1%), CD30 (3,1±1,4%), and CD15 (1,2±0,5%), with a significant decrease in the number of cells immunopositive for CD3 (*p*=0,03) and CD30 (*p*=0,01).

## Discussion

Antibacterial activity of AgNPs is determined by cascade of the reactions which is launched by penetration of positive ions of AgNPs through negatively-charged bacterial cell wall containing carboxylic, phosphate and amino groups. Static charge is created between two oppositely charged objects and forces the diffusion of nanoparticles even more which leads to disfunction of bacterial proteins, lipids and DNA. Ag+ binds with cell membrane proteins and creates sTablebonds with this membrane which leads to disfunction of proteins, decline of transmembrane ATP generation and indirect ion transport (13). Also ribosomes lose their structure and because of this translation processes and protein synthesis are inhibited (14).

AgNPs produce a lot of active forms of oxygen and free radicals (hydrogen peroxide, singlet oxygen, superoxide anion, hydroxyl radical, hypochlorous acid) and at the same time reduce expression of antioxidant enzymes (glutathione, superoxide dismutase, catalase). Dehydrogenase of mitochondrial electron transport chain is inactivated and this leads to amplification of lipid peroxidation, depletion of enzyme systems in cells, DNA damages (not only because of lipid peroxidation but also due to Ag+ intercalation between pairs of purine and pyrimidine bases which destroys hydrogen bonds, oppressing growth and vital activity of cells. (15-18).

According to recent researches VEGF (Vascular endothelial growth factor) level is increasing during inflammation. VEGF enhances antigen’s sensitisation and provides leak of proteins to the extravascular space, so T-helper cells come to this area and secrete proinflammatory cytokines such as IL-4, IL-5, IL-9, IL-13. VEGF and IL-1b stimulate endothelial permeability via Src-kinase phosphorylation. AgNPs directly block phosphorylation and inactivate Src-kinase, so endothelial permeability decreases, the level of proinflammatory cytokines lowers and cell elements stop migrating to the inflamed area (19).

The obtained results indicate a sufficient antimicrobial activity of the polylactide membrane coating with silver nanoparticles derived from a colloidal solution, which is consistent with the data of previous studies on the effectiveness of membranes and suture material coating with silver nanoparticles obtained via different methods (11,20). A lower proportion of CD3+ and CD30+ cells in the experimental group demonstrates a decrease in the activity of the inflammatory response to a foreign body, especially by activated lymphocytes. The migration activity of macrophages and neutrophils was also reduced in the experimental group, although the decrease in the level of CD15 immunopositivity did not reach statistical significance. These results along with a decrease in the severity of fibrosis represented by organized capsule formation indicate the suppression of the inflammatory response at all stages of its development.

Thus, the colloidal silver solution as a source of silver nanoparticles for polylactide membranes coating showed potential as an antimicrobial and anti-inflammatory agent.
